# Treatment and prognosis study of spontaneous rupture hemorrhage in hepatocellular carcinoma: Recommendations for adding the A1 stage to the BCLC staging system

**DOI:** 10.1002/cam4.6952

**Published:** 2024-05-16

**Authors:** Qingqiang Ni, Hongtao Jia, Yazhou Zhang, Jun Lu, Hong Chang

**Affiliations:** ^1^ Department of Hepatobiliary Surgery Shandong Provincial Hospital Affiliated to Shandong First Medical University Jinan Shandong China; ^2^ Department of Pathology Shandong Provincial Hospital Affiliated to Shandong First Medical University Jinan Shandong China

**Keywords:** BCLC stage A, BCLC stage A1, BCLC staging system, hepatocellular carcinoma, overall survival, recurrence‐free survival, spontaneous rupture hemorrhage

## Abstract

**Background:**

The Barcelona Clinic Liver Cancer (BCLC) staging system is an internationally recognized clinical staging system for hepatocellular carcinoma (HCC). However, this staging system does not address the staging and surgical treatment strategies for patients with spontaneous rupture hemorrhage in HCC. In this study, we aimed to investigate the prognosis of patients with BCLC stage A undergoing liver resection for HCC with spontaneous rupture hemorrhage and compare it with the prognosis of patients with BCLC stage A undergoing liver resection without rupture.

**Methods:**

Clinical data of 99 patients with HCC who underwent curative liver resection surgery were rigorously followed up and treated at Shandong Provincial Hospital from January 2013 to January 2023. A retrospective cohort study design was used to determine whether the presence of ruptured HCC (rHCC) is a risk factor for recurrence and survival after curative liver resection for HCC. Prognostic comparisons were made between patients with ruptured and non‐ruptured BCLC stage A HCC (rHCC and nrHCC, respectively) who underwent curative liver resection.

**Results:**

rHCC (hazard ratio [HR] = 2.974, [*p*] = 0.016) and tumor diameter greater than 5 cm (HR = 2.819, *p* = 0.022) were identified as independent risk factors for overall survival (OS) after curative resection of BCLC stage A HCC. The postoperative OS of the spontaneous rupture in the HCC group (Group I) was shorter than that in the BCLC stage A group (Group II) (*p* = 0.008). Tumor invasion without penetration of the capsule was determined to be an independent risk factor for recurrence‐free survival (RFS) after liver resection for HCC (HR = 2.584, *p* = 0.002).

**Conclusion:**

HCC with concurrent spontaneous rupture hemorrhage is an independent risk factor for postoperative OS after liver resection. The BCLC stage A1 should be added to complement the current BCLC staging system to provide further guidance for the treatment of patients with spontaneous rupture of HCC.

## 1 INTRODUCTION

Globally, liver cancer ranks sixth in incidence among all cancers and fourth in cancer‐related mortality.^1^ Spontaneous rupture hemorrhage in hepatocellular carcinoma (HCC) is a severe complication of primary liver cancer. In some Asian regions, the incidence of HCC with concurrent rupture reaches is as high as 10%^2^ and the mortality rate exceeds 25%.^3^, ^4^, ^5^, ^6^, ^7^, ^8^ However, current research on whether HCC rupture affects overall patient survival and increases the risk of tumor recurrence presents conflicting results. Some studies suggest that spontaneous HCC rupture is a prognostic risk factor,^9^, ^10^, ^11^, ^12^, ^13^ whereas others argue that HCC rupture does not affect patient outcomes.^14^, ^15^, ^16^, ^17^ The clinical staging and optimal treatment strategies for patients with HCC complicated by rupture remain unclear, and further investigation is needed to assess the potential risk of intraperitoneal dissemination affecting prognosis.

Currently, the Barcelona Clinic Liver Cancer (BCLC) and TNM staging systems are the most widely used methods for staging and treating liver cancer. In the 8th edition of the American Joint Committee on Cancer TNM staging system, liver cancer complicated by rupture, due to its ability to penetrate the visceral peritoneum, is classified as T4 stage.^18^ However, owing to the clinical challenge of spontaneous rupture hemorrhage in liver cancer, the internationally adopted BCLC staging system for liver cancer does not provide explicit guidance.

China has one of the highest HCC incidence rates globally, making it a country with a notable prevalence of this disease. Rupture of HCC poses a significant clinical challenge for hepatobiliary surgeons and is associated with high mortality rates. Patients with ruptured HCC (rHCC) may be at a risk of tumor dissemination beyond the liver. However, a majority of these patients initially present with severe abdominal pain as their primary symptom with an abrupt onset. Furthermore, many patients lack pathological evidence of extrahepatic metastasis during the perioperative period. Consequently, they retain the potential for curative resection, which is not entirely equivalent to BCLC stage C, in which definite intra‐abdominal liver cancer lesions with extra‐liver metastases are present. Thus, investigating whether patients with spontaneous HCC rupture benefit from liver resection has significant clinical relevance. In this study, we compared the postoperative overall survival (OS) and recurrence‐free survival (RFS) of patients with BCLC stage A with spontaneous rupture of HCC with those without rupture. The objective of this study was to further elucidate the impact of spontaneous rupture hemorrhages on the survival of patients with BCLC stage A after curative resection, providing valuable insights for the ongoing refinement of the BCLC staging system.

## 2 DATA AND METHODS

### 2.1 General information

A retrospective cohort design was used in this study. Data were collected from patients who underwent curative liver resection surgery for HCC at the Department of Hepatobiliary Surgery, Shandong Provincial Hospital, between January 2013 and January 2023 and were rigorously followed up and treated in our hospital. After applying strict exclusion criteria, 99 patients were included in the study. Among them, 89 were males (89.9%) and 10 females (10.1%), with a male‐to‐female ratio of 8.9:1. The mean age of the participants was (57.06 ± 10.01) years. The diagnosis of HCC was confirmed based on the postoperative pathological examination. The diagnosis of liver cancer rupture was made through a combination of clinical history, preoperative computed tomography (CT), ultrasonography, magnetic resonance imaging (MRI), intraoperative findings and further confirmation was made through postoperative pathology. This study was approved by the Ethics Committee of Shandong Provincial Hospital and was conducted in accordance with the principles of the Declaration of Helsinki. All patients and their families provided informed consent prior to the procedure.

### 2.2 Inclusion and exclusion criteria

#### 2.2.1 Inclusion criteria

(1) First‐time diagnosis confirmed of HCC confirmed via postoperative pathology. (2) No history of other systemic malignancies. (3) Complete intraoperative resection of visible nodules with postoperative pathological confirmation of negative margins under the microscope and the absence of microvascular invasion around the tumor. (4) A single tumor with a diameter >2 cm meeting the 2022 BCLC A‐stage recommendations for preferred surgical resection. (5) Good physical condition with an ECOG score ≤1 and Child–Pugh classifications A–B. (6) In addition to the above conditions, the inclusion criteria for the liver cancer rupture group included: (1) Initial diagnosis of primary liver cancer with rupture and surgical resection performed within 1 week of diagnosis. (2) No visible metastatic nodules observed during intraoperative exploration. If adhesions to the peritoneum or blood staining are present, concurrent resection is required, and pathological evidence does not show tumor spread.

#### 2.2.2 Exclusion criteria

(1) Postoperative pathology confirming non‐HCC, such as intrahepatic cholangiocarcinoma or mixed‐type liver cancer. (2) Perioperative mortality. (3) Patients with a number of intrahepatic tumor lesions ≥2 on preoperative imaging or intraoperative findings, for both BCLC stages A and B patients. (4) BCLC stages C and D. (5) Patients with incomplete clinical data, lost to follow‐up, noncompliance with regular postoperative follow‐ups in our hospital, or those who did not continue treatment in our hospital after disease recurrence or progression. (6) Exclusion criteria specific to the liver cancer rupture group included: (1) Nonspontaneous rupture hemorrhage patients with a history of significant trauma or other inducing factors. (2) Preoperative presence of severe portal hypertension and/or history of local interventional or systemic treatment. (3) Postoperative pathology indicating proximity to the surgical margin and/or presence of vascular infiltration.

### 2.3 Grouping

The 99 patients with HCC included in the study who underwent liver resection surgery were divided into two groups: spontaneously rHCC (Group I) consisting of 32 patients and BCLC stage A patients without rupture who were recommended for surgical resection (Group II) consisting of 67 patients.

### 2.4 Definition of resectable rHCC

The resectable rHCC group includes patients in good physical condition and no history of cardiac, pulmonary, or renal dysfunction, with a Child–Pugh classification of A–B. Hemodynamic stability is achieved after fluid resuscitation, blood transfusion, and other necessary treatments. Willingness to undergo liver resection surgery. Preoperative assessment to confirm the feasibility of achieving a safe R0 resection. Adequate remaining liver volume (non‐cirrhotic liver patients ≥ 30%, cirrhotic liver patients ≥ 40%). Preoperative imaging and intraoperative exploration revealing a single ruptured lesion. No evidence of metastatic lesions at the rupture site or peritoneal cavity. Rapid pathological confirmation of the absence of tumor components in the surrounding inflammatory tissue and encapsulation of the peritoneum. Absence of significant contraindications to surgery.

### 2.5 Definition of curative liver resection

Curative liver resection includes patients with no history of cardiac, pulmonary, or renal dysfunction, and no significant surgical contraindications. The surgical approach and extent of liver resection are determined based on the tumor size, location, and liver functional reserve. Curative liver resection must meet the following criteria: (1) preoperative assessment confirms the feasibility of achieving safe R0 resection with no residual tumor. (2) Adequate remaining liver volume (non‐cirrhotic patients ≥ 30%, cirrhotic patients ≥ 40%). (3) Pathological examination reveals negative surgical margins. (4) For patients with rHCC, concurrent resection of the affected greater omentum and adjacent tissues along with peritoneal lavage, is required to achieve curative resection. Preoperative liver function assessment is based on the Child–Pugh scoring system. Diagnoses are evaluated by three experienced pathologists; in cases of disagreement in their conclusions, a final diagnosis was reached through consultation with all three experts.

### 2.6 Observational parameters

(1) Preoperative examination results: liver function, tumor markers, and imaging findings. (2) surgical treatment details: surgical approach, duration, and intraoperative blood transfusion. (3) postoperative pathological examination: tumor differentiation grade and distance from the tumor to the surgical margin. (4) postoperative complications: intraperitoneal bleeding, abdominal infections, pleural effusion, etc. (5) patient follow‐up outcomes: time of tumor recurrence and postoperative survival time.

### 2.7 Follow‐up visits

Patient‐related data were collected by follow‐up with patients through telephone calls, outpatient visits, and readmission to assess their prognosis. Outcomes included OS and RFS. OS was defined as the time from surgery to either the patient's death or the end of the follow‐up period, whereas RFS was defined as the time from surgery to the first documented recurrence. Recurrence was primarily diagnosed based on imaging examinations such as ultrasonography (B‐mode), CT, MRI, and local transarterial chemoembolization (TACE), or confirmed by pathology through secondary resection or percutaneous biopsy. The follow‐up period began at the time of curative liver resection and ended on January 30, 2023, or at the time of patient death, loss to follow‐up, or if patients did not return for an outpatient visit within 3 months, their phone was unreachable, or they were admitted to another hospital for treatment, they were considered lost to follow‐up.

### 2.8 Statistical analysis

All the statistical analyses were performed using SPSS version 26 (IBM Corp, Armonk, NY, USA). Normally distributed continuous data are presented as mean ± standard deviation, and the independent sample *t*‐test was used to compare the two groups. Non‐normally distributed continuous data were expressed as medians and interquartile ranges, and between‐group comparisons were performed using non‐parametric tests. Categorical data are presented as frequencies and percentages and were compared using the chi‐square test or Fisher's exact test. Kaplan–Meier curves were used to depict the OS and RFS rated for both groups, and the log‐rank test was used to compare the OS and RFS rates between the two groups. A multivariate Cox regression model was utilized to analyze the correlation between liver cancer rupture and patient prognosis (including OS and RFS) while exploring other risk factors affecting the overall patient prognosis. All tests were two‐tailed, and a *p*‐value of less than 0.05 was considered statistically significant.

## 3 RESULTS

### 3.1 Statistical description of general characteristics in the 99 HCC patients undergoing radical resection

#### 3.1.1 Overall characteristics

There were 89 male patients (89.9%) with an average age of 57.06 ± 9.704 years. Of the total number of patients, 82 (82.8%) had a history of hepatitis B, 28 (28.3%) had a history of hypertension, eight (8.1%) had a history of diabetes, five (5.1%) had a history of coronary heart disease, and three (3.0%) had a family history of liver cancer. The average length of hospital stay was 12.53 ± 4.797 days.

#### 3.1.2 Preoperative liver function indicators

Total bilirubin: 20.3 ± 13.7 μmol/L, albumin (Alb): 40.0 ± 5.2 g/L, alanine aminotransferase: 32 (24, 55) U/L, and aspartate aminotransferase: 37 (26, 61) U/L.

#### 3.1.3 Tumor characteristics

The tumor diameter was 56.4 ± 31.3 mm. A total of 28 patients (28.3%) had poorly differentiated tumors, and 49 (49.5%) tested positive for alpha‐fetoprotein (AFP).

#### 3.1.4 Surgical information

The average surgical duration was 167.4 ± 61.3 min. Blood transfusions were required in 24 patients (24.2%), and 64 (64.6%) had underlying liver cirrhosis.

#### 3.1.5 Postoperative complications

In group I, one patient (3.1%) had pleural effusion, two (6.3%) had abdominal effusion, two (6.3%) had intra‐abdominal bleeding, one (3.1%) had abdominal infection, one (3.1%) had pulmonary infection, one (3.1%) had bile duct fistula, and one (3.1%) developed deep vein thrombosis in both lower limbs. In group II, three patients (4.5%) had pleural effusion, 6 (4.5%) had abdominal effusion, 3 (4.5%) had intra‐abdominal bleeding, 2 (3.0%) had abdominal infection, 2 (3.0%) had pulmonary infection, and 3 (4.5%) developed bile duct fistulas.

### 3.2 Comparative analysis of general and clinical characteristics between the two groups of patients

Group I comprised 32 individuals, including 31 males (96.9%). The median age was 56.0 (51.3, 61.0) years. Group II comprised 67 individuals, including 58 males (86.6%). The median age was 59.0 (50.0, 65.0) years. There were no statistically significant differences in the general or clinical characteristics, including age, sex, history of diabetes, history of hypertension, history of coronary heart disease, history of abdominal surgery, AFP positivity, liver cirrhosis, preoperative albumin level, preoperative bilirubin level, and maximum tumor diameter (*p* > 0.05), as shown in Table 1.

**TABLE 1:** Analysis of general and clinical data.

Variable	Group I (*n* = 32)	Group II (*n* = 67)	*p*‐value
Age (years)	56.0 (51.3, 61.0)	59.0 (50.0, 65.0)	0.310
Male	31 (96.9%)	58 (86.6%)	0.160
Post‐recurrence survival with abdominal metastasis	3 (9.4%)	4 (6%)	0.678
History of diabetes	4 (12.5%)	4 (6%)	0.269
History of hypertension	9 (28.1%)	19 (28.4%)	0.981
History of coronary heart disease	2 (6.3%)	3 (4.5%)	0.657
History of abdominal surgery	1 (3.1%)	10 (14.9%)	0.081
Invasion of the liver capsule	5 (15.6%)	8 (11.9%)	0.612
AFP‐positive tumors	15 (46.9%)	37 (55.2%)	0.437
Cirrhosis	23 (71.9%)	41 (61.2%)	0.298
TBiL (μmol/L)	24.02 ± 15.97	18.53 ± 12.29	0.092
Albumin (g/L)	38.76 ± 6.29	40.60 ± 4.59	0.145
Hepatitis B	28 (87.5%)	57 (85.1%)	0.746
Max tumor diameter (cm)	55.0 (39.0)	50.0 (30.0)	0.054
HBeAG	4 (12.5%)	8 (11.9%)	0.612
Blood transfusion	13 (40.6%)	11 (16.4%)	0.009
Surgery time (min)	180.0 (150.0, 210.0)	156.0 (120.0,1 80.0)	0.052
Position			
Left	12 (37.5%)	14 (20.9%)	0.070
Caudate	2 (6.3%)	1 (1.5%)	
Right	18 (56.3%)	52 (77.6%)	
Resection range			
Half liver	3 (9.4%)	6 (9.0%)	0.606
Limited	29 (91.6%)	61 (91.0%)	

Abbreviation: AFP, alpha‐fetoprotein.

### 3.3 Analysis of factors affecting OS in patients after HCC radical resection

Cox univariate analysis results showed that liver cancer rupture and maximum tumor diameter were risk factors affecting the OS of HCC patients after radical liver resection (*p* < 0.05). After incorporating meaningful prognostic factors from the univariate analysis into the Cox multivariate analysis, the results showed that liver cancer rupture (HR = 2.974, *p* = 0.016) and maximum tumor diameter (HR = 2.819, *p* = 0.022) were independent risk factors for OS in HCC patients after radical resection, as shown in Table 2.

**TABLE 2:** Liver rupture and tumor diameter as independent risk factors for postoperative overall survival.

Variable	Univariate analysis		Multivariate analysis	
	HR (95% CI)	*p*‐value	HR (95% CI)	*p*‐value
Age (years)				
>60	1.311 (0.52–3.258)	0.560		
≤60				
Sex				
Male	1.594 (0.211–12.047)	0.651		
Female				
TBiL (μmol/L)				
>20	0.942 (0.395–2.248)	0.894		
≤20				
Alb (g/L)				
≤35	0.585 (0.216–1.587)	0.292		
>35				
ALT (U/L)				
≤40	0.973 (0.409–2.318)	0.951		
>40				
AFP (μg/L)				
>20	1.052 (0.421–2.630)	0.913		
≤20				
Cirrhosis				
Yes	2.143 (0.805–5.704)	0.127		
No				
Ruptured HCC				
Yes	3.156 (1.303–7.647)	0.011	2.974 (1.222–7.239)	0.016
No				
HBeAG				
Yes	0.657 (0.152–2.846)	0.572		
No				
Tumor diameter				
>5 cm	2.999 (1.233–7.296)	0.015	2.819 (1.159–6.856)	0.022
≤5 cm				
Intraoperative blood transfusion				
Yes	0.672 (0.270–1.672)	0.392		
No				
Surgical approach				
Laparotomy	0.871 (0.524–1.449)	0.595		
Laparoscopic surgery				
Invasion of the membrane	0.831 (0.271–2.544)	0.746		
Degree of tumor differentiation				
Low differentiation	1.668 (0.610–4.560)	0.319		
High and medium differentiation				

Abbreviation: AFP, alpha‐fetoprotein.

### 3.4 Comparison of survival rates between Group I and Group II patients after curative resection

**FIGURE 1:**
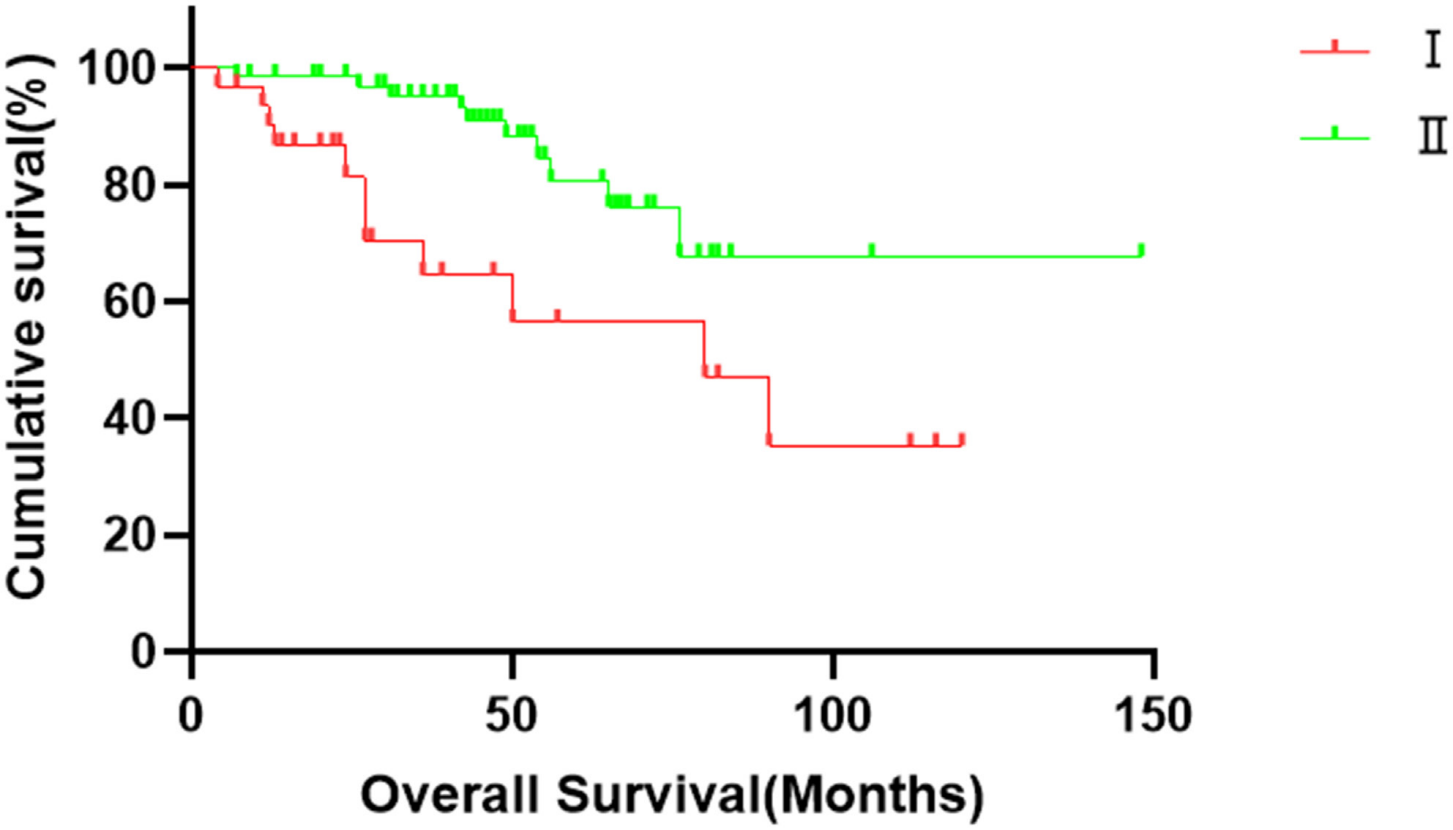
Overall survival curves of the two study groups. There is a statistically significant difference in overall survival between Groups I and II (*p* < 0.05). Specifically, Group I exhibited a shorter overall survival than Group II (*p* = 0.008).

The median survival time for Group I was 80 months (95% confidence interval [CI]: 31.252–128.748), with 1‐, 3‐, and 5‐year OS rates of 90.2%, 64.6%, and 56.5%, respectively. Group II had 1‐, 3‐, and 5‐year OS rates of 98.5%, 95.1%, and 80.5%, respectively. The differences in the OS rates between the two groups was a statistically significant (*p* < 0.05), with Group I having a shorter OS rate than Group II (*p* = 0.008). The OS curves are shown in Figure 1.

### 3.5 Analysis of factors affecting RFS in patients after radical resection

The results of Cox univariate analysis demonstrated that tumor invasion of the liver capsule without penetration of the capsule was a risk factor for RFS in patients undergoing radical hepatectomy for HCC (*p* = 0.002). After incorporating the meaningful prognostic factors identified in the univariate analysis into the Cox multivariate analysis, the findings revealed that tumor invasion of the liver capsule without penetration of the capsule was an independent risk factor affecting RFS in patients who underwent radical hepatectomy for HCC (HR = 2.584, *p* = 0.002), as shown in Table 3.

**TABLE 3:** Tumor liver capsule invasion as an independent risk factor affecting recurrence‐free survival after liver cancer surgery.

Variable	Univariate analysis		Multivariate analysis	
	HR (95% CI)	*p*‐value	HR (95% CI)	*p*‐value
Age (years)				
>60	0.865 (0.542–1.379)	0.541		
≤60				
Sex				
Male Female	1.163 (0.578–2.340)	0.672		
TBiL (μmol/L)				
>20	1.264 (0.795–2.009)	0.322		
≤20				
Alb(g/L)				
≤35	1.493 (0.709–3.14)	0.292		
>35				
ALT (U/L)				
≤40	0.856 (0.534–1.371)	0.518		
>40				
AFP(μg/L)				
>20	0.946 (0.590–1.519)	0.820		
≤20				
Cirrhosis				
Yes	1.221 (0.759–1.964)	0.410		
No				
HBeAg				
Yes	1.724 (0.897–3.313)	0.098		
No				
Ruptured HCC				
Yes	1.111 (0.657–1.881)	0.694		
No				
Hb > 120				
Yes	0.634 (0.348–1.155)	0.137		
No				
Tumor diameter				
>5 cm	1.188 (0.752–1.875)	0.461		
≤5 cm				
Intraoperative blood transfusion				
Yes	1.175 (0.674–2.049)	0.568		
No				
Surgical approach				
Laparotomy	1.575 (0.525–4.731)	0.418		
Laparoscopic surgery				
Maximum tumor diameter >5(cm)	1.188 (0.752–1.875)	0.461		
Invasion of the liver capsule	2.584 (1.403–4.759)	0.002	2.584 (1.403–4.759)	0.002
Degree of tumor differentiation				
Low differentiation	1.219 (0.754–1.971)	0.420		
High and medium differentiation				

### 3.6 Comparison of RFS after radical resection between Groups I and II

**FIGURE 2:**
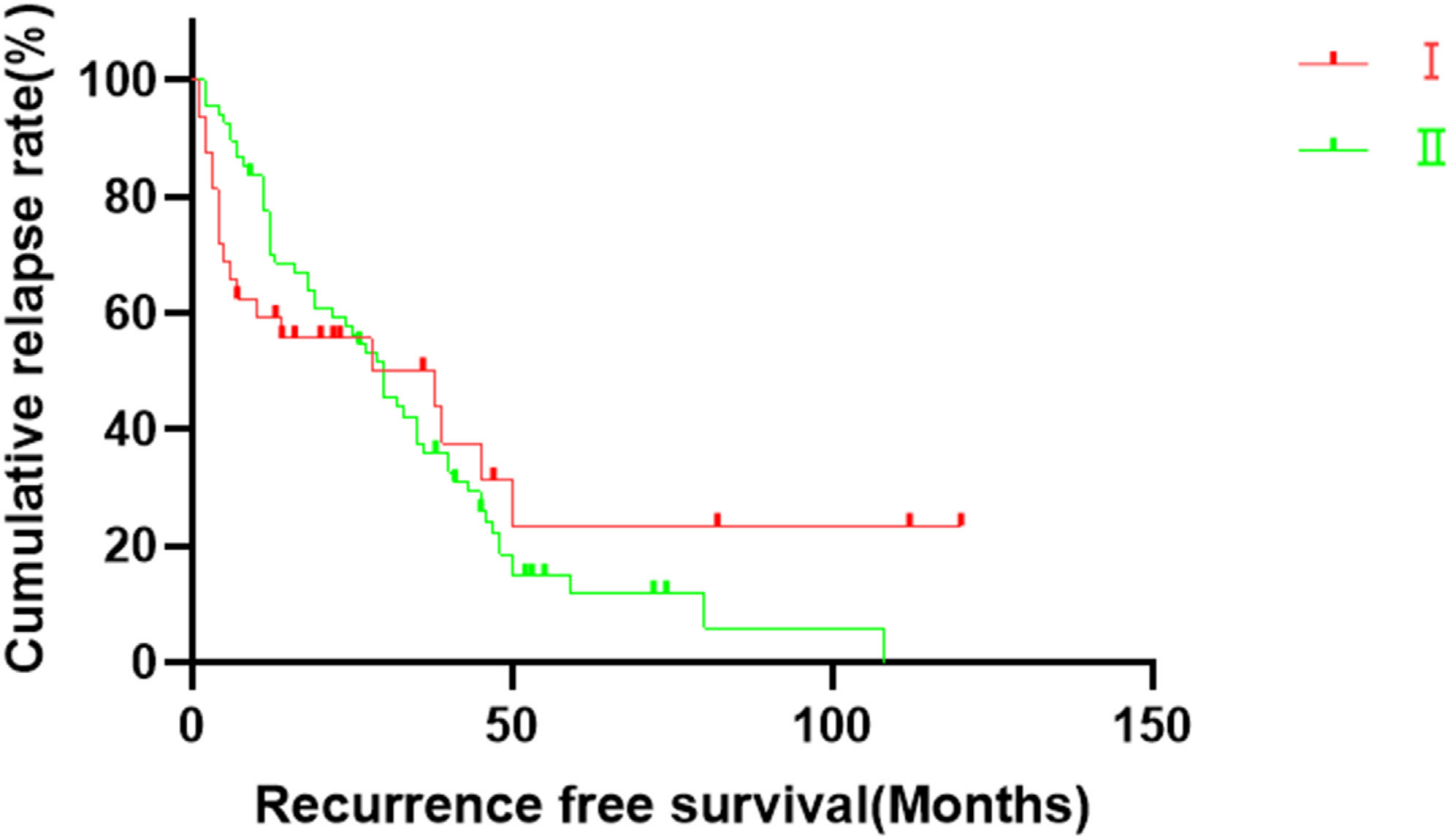
Recurrence‐free survival curves of the two study groups. There was no statistically significant difference in the recurrence‐free survival time between Groups I and II (*p* = 0.688).

The median RFS time in Group I was 38 months (95% CI: 0.0–78.6), with 1‐, 3‐, and 5‐year RFS rates of 59.2%, 50.2%, and 23.5%, respectively. In Group II, the median RFS rate was 30 months (95% CI: 24.2–36.7), with the 1‐, 3‐, and 5‐year RFS rates of 77.5%, 36.0%, and 11.8%, respectively. No statistically significant difference in RFS between the two groups was noted (*p* = 0.688). The RFS curves are shown in Figure 2.

## 4 DISCUSSION

Spontaneous rupture of a HCC is a severe and life‐threatening complication. Some patients who are generally in good condition and have good liver function experience early tumor rupture due to factors such as rapid tumor growth, external pressure, and exophytic growth. Currently, there are no unified guidelines for the classification and treatment of patients with ruptured liver cancers. Consequently, previous studies have used different selection criteria and monitoring mechanisms for these patients, leading to controversies regarding whether spontaneous rupture of HCC is an independent risk factor affecting patient prognosis.^3^, ^4^, ^19^, ^20^, ^21^ In this study, we found a statistically significant difference in the OS rates between the rHCC and nrHCC groups (*p* < 0.05). The rHCC group had worse OS than the nrHCC group (*p* = 0.011), whereas there was no statistically significant difference in RFS between the two groups (*p* = 0.688). These results are consistent with those of a study by JIN et al., which showed that although RFS was comparable (*p* = 0.049), patients with rHCC had a significantly worse OS than those with nrHCC (*p* < 0.001).^22^ Another study with a larger sample size demonstrated the significant impact of liver cancer rupture on both RFS and OS. However, in this study, differences in microvascular invasion were found between the groups, the number of tumors were not specified, and the study participants included patients who underwent elective resection within 1 month.^13^ A prospective study indicated that patients with a single tumor have favorable prognosis,^23^ while another study indicated that patients with rHCC who underwent immediate resection (<1 week) had lower recurrence rates and fewer peritoneal metastases than those who underwent elective resection.^24^ Early resection within 1 week of diagnosis is beneficial. In addition to its hemostatic effect, liver resection completely removes the primary lesion, offering excellent therapeutic efficacy against ruptured liver cancer with rupture.^15^, ^20^, ^24^, ^25^ Therefore, including patients with liver cancer rupture in the BCLC stage C category who primarily receive localized TACE, molecular targeted therapy, and immunotherapy may result in some early stage patients losing the opportunity for curative resection.

In this study, we found that tumor volume was associated with OS, and patients with a maximum tumor diameter <5 cm had longer survival times. With continuous improvements in the technical capabilities of various liver cancer centers, the resection of large liver cancers is gradually becoming feasible. However, larger tumor diameters may be associated with a higher risk of positive margins.^26^ Research has shown that patients with small‐volume rHCC who undergo curative resection can achieve a prognosis similar to that of nrHCC patients.^15^, ^27^ Therefore, based on the characteristics of BCLC stage A tumors, we recommend that early surgical resection should be considered as the primary treatment option for patients with a single tumor with a diameter >2 cm, who have spontaneously ruptured without evidence of peritoneal implantation and who have good liver function (Child–Pugh score) and ECOG performance status.

**FIGURE 3:**
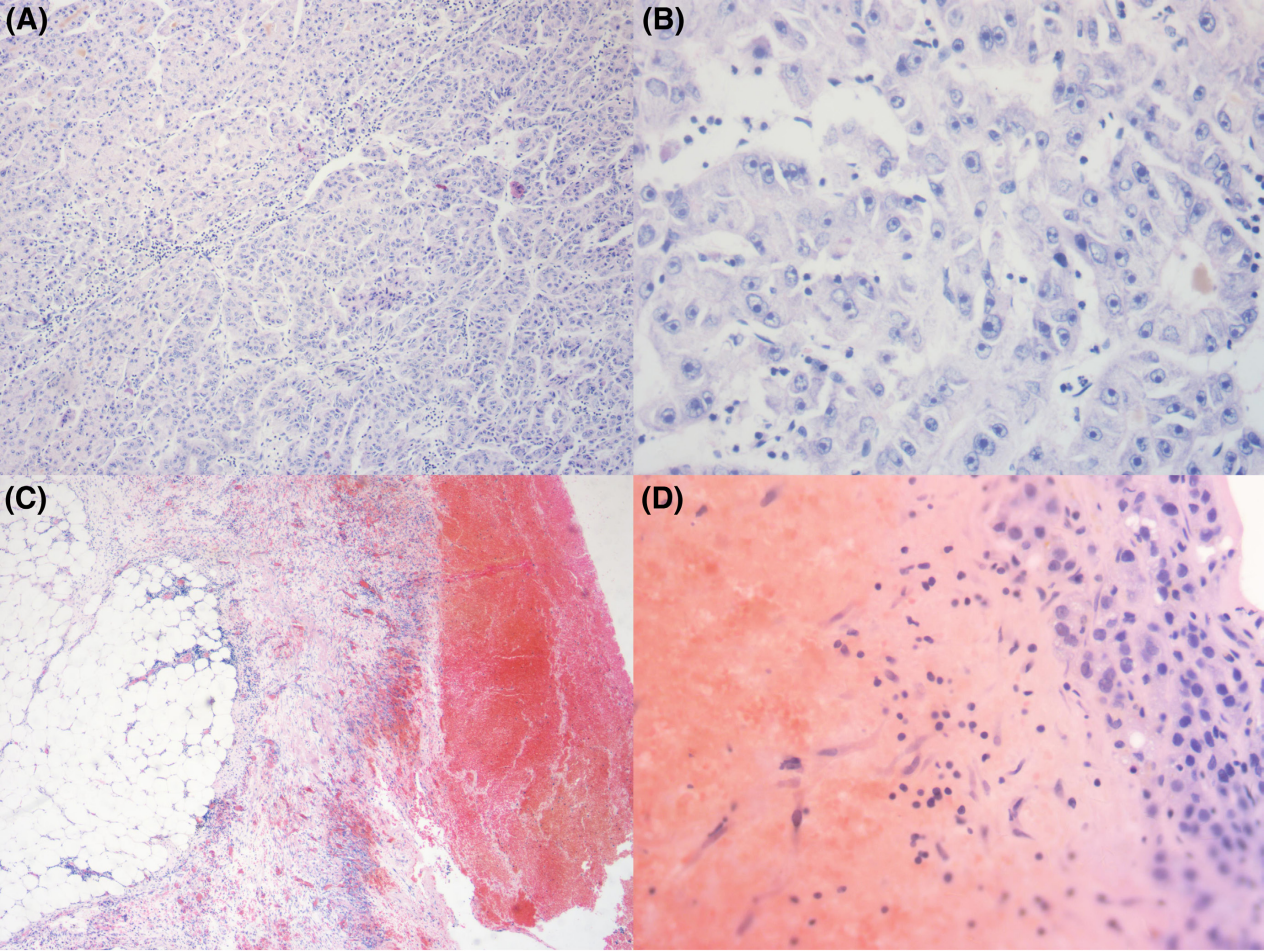
A section of hepatocellular carcinoma (HCC) tissues with most of the tumor cells arranged in a pseudoglandular pattern, with obvious cellular atypia and clear nucleoli. (A). Magnification 100×. (B). Magnification 400×. Hemorrhage and reactive hyperplasia of fibrous tissues are present on the surface of the omentum, and HCC is present in the hemorrhagic foci, thus suggesting tumor spread and metastasis. (C). Magnification 40×. (D). Magnification 400×.

However, according to the previous literature, unlike late‐stage liver cancer, patients with rHCC seem to experience peritoneal implantation, which occurs more often in the early stages.^28^, ^29^ Due to incomplete tumor capsules in rHCC, tumor components enter the peritoneal cavity, and the few surviving tumor cells can lead to undetectable intraoperative peritoneal dissemination.^29^, ^30^, ^31^, ^32^, ^33^, ^34^ Therefore, it is essential to reduce the potential risk of peritoneal spread of rHCC compared to that of nrHCC. Our research team also identified patients with evidence of peritoneal implantation during surgery. In the four excluded patients, 10–15 days after the onset of rupture, exploration revealed involvement of the tissues surrounding the ruptured tumor, including the greater omentum, diaphragm, and intestinal wall nodules. Postoperative pathology confirmed the presence of peritoneal tumor cell dissemination (Figure 3). This retrospective analysis included 32 patients with spontaneous rupture who underwent curative resection for early surgical management of liver cancer over the past decade. Based on the assessment of liver function (Child–Pugh score), ECOG performance status, and imaging tumor burden, 32 patients with single liver cancer, >2 cm in diameter underwent curative resection. All patients in the liver cancer rupture group underwent surgery within 1–7 days of liver cancer rupture. The intraoperative use of distilled water to lavage the peritoneal cavity has been reported to improve patient outcomes.^35^ This method effectively removes macroscopically invisible tumor cells, reducing the risk of peritoneal implantation.^24^, ^36^, ^37^ Our study did not find any differences in the postoperative peritoneal spread between the two groups. There was no statistically significant difference in the RFS rate between patients with liver cancer that ruptured spontaneously and patients with BCLC stage A liver cancer that had not ruptured (*p* = 0.688). Therefore, meticulous intraoperative exploration is needed for an effective approach to ensure the absence of macroscopically visible implant nodules, along with surgical curative resection and abdominal irrigation with hot distilled water.

Despite timely surgery and rigorous intraoperative preventive measures, the long‐term survival of these patients remains a concern. Some studies have shown that although the short‐term prognosis of patients with liver cancer that ruptures spontaneously at BCLC stage A is superior to that of TACE, the long‐term prognosis is comparable to that of TACE.^38^ Therefore, for liver cancer with spontaneous rupture, given the high risk of tumor implantation, poorer biological behavior compared to that of non‐ruptured liver cancer, and the recommended primary treatment of early surgical resection, strict postoperative follow‐up and monitoring of peritoneal implantation in high‐risk patients are mandatory. Timely and effective immunotherapies and targeted therapies are recommended for patients with BCLC progression stage. This approach aligns with the concept of “treatment stage migration” in the new version of BCLC (2022), involving early resection for select patients and switching to systemic treatment as needed.^39^

**FIGURE 4:**
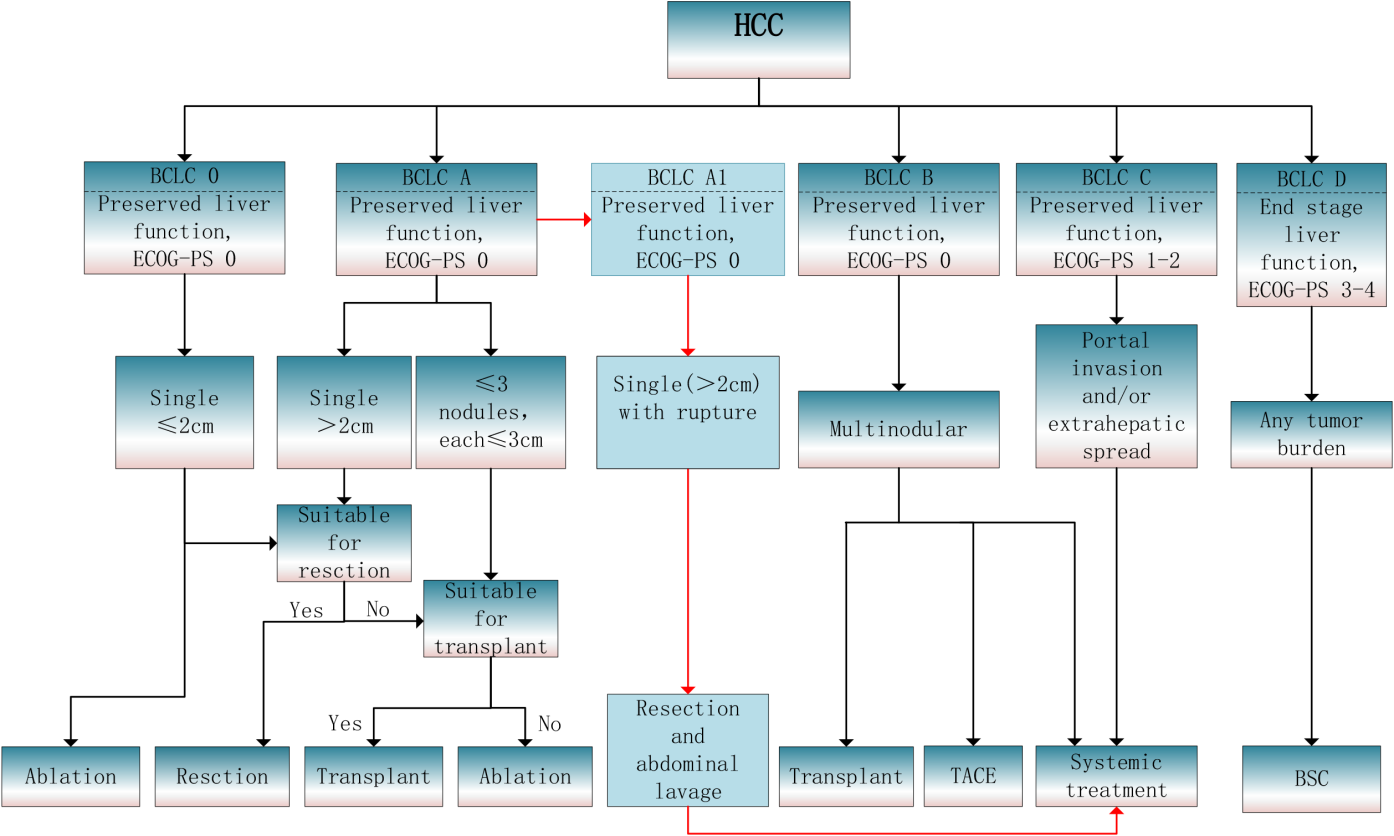
Flowchart of the newly added BCLC A1 staging diagnostic and treatment protocol. HCC, hepatocellular carcinoma; BCLC, Barcelona Clinic Liver Cancer; BSC, best supportive care; ECOG‐PS, Eastern Cooperative Oncology Group‐performance status; TACE, transarterial chemoembolisation.

Currently, the BCLC staging system does not provide clear guidance on the clinical issues of liver cancer with spontaneous rupture, nor does it offer uniform criteria for evaluating the severity of liver cancer rupture and specific treatment approaches. Based on our research results, patients with a single liver tumor >2 cm who experienced spontaneous rupture (median survival of 80 months) had a much longer life expectancy than that suggested for BCLC B stage patients (>30 months) and was similar to the life expectancy of BCLC A stage patients (>60 months) mentioned in the BCLC guidelines. We believe that ruptured liver cancer carries a high risk of distant metastasis, rendering liver transplantation unsuitable for these patients. Therefore, this specific patient group should be included in the new BCLC staging system, based on the existing system. In conclusion, this study is the first to propose the inclusion of patients with liver cancer with spontaneous rupture into the current BCLC liver cancer staging system. For patients with a single liver tumor >2 cm who have experienced spontaneous rupture, without evidence of peritoneal implantation, and with good liver function (Child–Pugh score) and ECOG performance status, we recommend classifying them as BCLC stage A1, with emphasis on early surgical resection and intraoperative distillation with hot water. Postoperative “treatment stage migration” should include systemic drug therapy for C stage patients, as shown in Figure 4.

Given that the population of patients with non‐ruptured BCLC stage A HCC had a better prognosis, with a death rate of less than half, this study was unable to calculate the median survival time for these patients. This single‐center retrospective cohort study included patients treated at the Hepatobiliary Surgery Department of Shandong Provincial Hospital. Inevitable biases may exist, and further verification through multicenter clinical studies with larger sample sizes is required.

## AUTHOR CONTRIBUTIONS

**Qingqiang Ni:** Formal analysis; writing – original draft; writing – review and editing; funding acquisition. **Hongtao Jia:** Data curation; writing – original draft. **Yazhou Zhang**: Investigation; methodology; resources. **Jun Lu**: Supervision; writing – review and editing. **Hong Chang**: Conceptualization; data curation; funding acquisition; project administration; writing – review and editing.

## FUNDING INFORMATION

This study was supported by Shandong Provincial Natural Science Foundation, Grant/Award Number: ZR2020MH054; Taishan Scholars Program of Shandong Province of China, Grant/Award Number: tsqn202306374.

## CONFLICT OF INTEREST STATEMENT

The authors of this manuscript have no conflicts of interest to disclose.

## ETHICS STATEMENT

The study was approved by the Ethics Committee of Shandong Provincial Hospital and was registered at Clinical Trials Registry (ChiCTR2200066839). Due to its retrospective nature, the study did not contain recorded information about patients' privacy. Therefore, patients' informed consent could be exempted.

## Data Availability

The raw data supporting the conclusions of this article will be available upon request from the corresponding author.
